# Ultrasound-Guided Aspiration and Dextrose Prolotherapy for a Triangular Fibrocartilage Complex Ganglion Cyst in an Avid Golfer: A Case Report

**DOI:** 10.7759/cureus.101874

**Published:** 2026-01-19

**Authors:** Yonghyun Yoon, Stephen Cavallino, Teinny Suryadi, Anwar Suhaimi, Jonghyeok Lee, King Hei Stanley Lam

**Affiliations:** 1 Orthopedic Surgery, Hallym University Kangnam Sacred Heart Hospital, Seoul, KOR; 2 Orthopedics, Incheon Terminal Orthopedic Surgery Clinic, Incheon, KOR; 3 Orthopedics, International Academy of Regenerative Medicine, Incheon, KOR; 4 Prolotherapy and Regenerative Medicine, European School of Prolotherapy, Rome, ITA; 5 Physical Medicine and Rehabilitation, Synergy Clinic, Jakarta, IDN; 6 Physical Medicine and Rehabilitation, Hermina Podomoro Hospital, Jakarta, IDN; 7 Rehabilitation Medicine, University Malaya Medical Centre, Kuala Lumpur, MYS; 8 Rehabilitation Medicine, University of Malaya, Kuala Lumpur, MYS; 9 Neurosurgery, Barun Neurosurgery Clinic, Cheongju, KOR; 10 Faculty of Medicine, The Chinese University of Hong Kong, New Territories, HKG; 11 Faculty of Medicine, The University of Hong Kong, Hong Kong, HKG; 12 Board of Clinical Research, Hong Kong Institute of Musculoskeletal Medicine, Kowloon, HKG

**Keywords:** ganglion cyst, prolotherapy, tfcc, triangular fibrocartilage complex, ultrasound-guided intervention, wrist pain

## Abstract

The triangular fibrocartilage complex (TFCC) is a key stabilizer of the ulnar side of the wrist, and ganglion cysts arising primarily from the TFCC are uncommon, with no clear consensus on optimal management.

We report the case of a 63-year-old right-handed male businessman and avid golfer, whose work required regular business golf rounds, presenting with several months of persistent left ulnar-sided wrist pain that worsened during golf swings when not using a brace. Physical examination revealed a positive ulnar fovea sign and pain with grip strength testing. Radiographs demonstrated neutral ulnar variance, and computed tomography showed no evidence of fracture or significant arthritic changes. Ultrasonography identified a multiseptated ganglion cyst adjacent to the TFCC. Under ultrasound guidance, the cyst was aspirated, followed by prolotherapy using 10% dextrose injected into the TFCC enthesis. The patient subsequently underwent a structured rehabilitation program including physical therapy and isometric exercises. After five sessions of prolotherapy at two-week intervals, his pain resolved completely, and he was able to maintain his work-related golf schedule without discomfort.

This case suggests that ganglion cysts may occur secondary to TFCC pathology and underscores the value of ultrasound not only for diagnosis but also for guiding minimally invasive interventions. Ultrasound-guided aspiration combined with prolotherapy may represent an effective, joint-preserving treatment option for TFCC-associated ganglion cysts, potentially avoiding the need for more invasive surgical procedures in selected patients.

## Introduction

The triangular fibrocartilage complex (TFCC) is a crucial structure comprising ligaments and fibrocartilage that stabilizes the ulnar side of the wrist, supports the distal radioulnar joint (DRUJ), the articulation between the distal radius and ulna, and acts as a primary load bearer. It is a common source of ulnar-sided wrist pain. Injuries to the TFCC are classified traumatically (Palmer Class 1) or degeneratively (Palmer Class 2), guiding treatment strategies that range from conservative management to surgical repair [1].

While wrist arthroscopy remains the diagnostic gold standard for TFCC injuries, magnetic resonance arthrography (MRA) is widely used in clinical practice as a less invasive imaging modality. In addition, high-resolution ultrasonography has emerged as a valuable, dynamic, and accessible tool for evaluating TFCC pathology [2]. Ganglion cysts associated with the TFCC are rare findings and are thought to arise from mucoid degeneration or as a communication from an underlying tear [3].

Standard treatments for symptomatic wrist ganglions include aspiration or surgical excision, but recurrence is common regardless of the approach [4]. Prolotherapy, an injection therapy that aims to stimulate tissue repair and strengthen ligamentous structures, has shown promise in managing various musculoskeletal conditions, including chronic wrist pain [5-7].

However, there is a scarcity of literature describing the use of ultrasound-guided aspiration combined with prolotherapy specifically for TFCC ganglion cysts. This case report details the successful management of such a cyst using this combined, minimally invasive approach.

## Case presentation

A 63-year-old right-handed male businessman and avid golfer, who played at least twice weekly for work-related business meetings, presented with a six-month history of persistent, dull aching pain on the ulnar aspect of his left wrist. The pain was exacerbated during his golf swing, especially when he did not use a supportive wrist brace. He denied any history of acute trauma, night pain, or morning stiffness. At presentation, his pain severity was rated as 7/10 on a numeric rating scale (NRS).

On physical examination, there was localized tenderness at the ulnar fovea (the soft spot between the ulnar styloid and flexor carpi ulnaris tendon), consistent with a positive ulnar fovea sign. The ballottement and grind tests reproduced the patient’s characteristic ulnar-sided wrist pain but did not demonstrate objective instability when compared with the contralateral wrist. The piano key sign was negative, indicating no clinically apparent DRUJ instability. Grip strength on the affected side was reduced to approximately 70% of the contralateral side as measured using a handheld dynamometer. The wrist demonstrated a full pain-free range of motion. No visible swelling or palpable mass was noted on inspection or palpation. Given that golf played a central role in his business activities and client relationships, he was highly motivated to pursue a treatment strategy that would allow early return to play and avoid prolonged immobilization or surgery.

**Figure 1:**
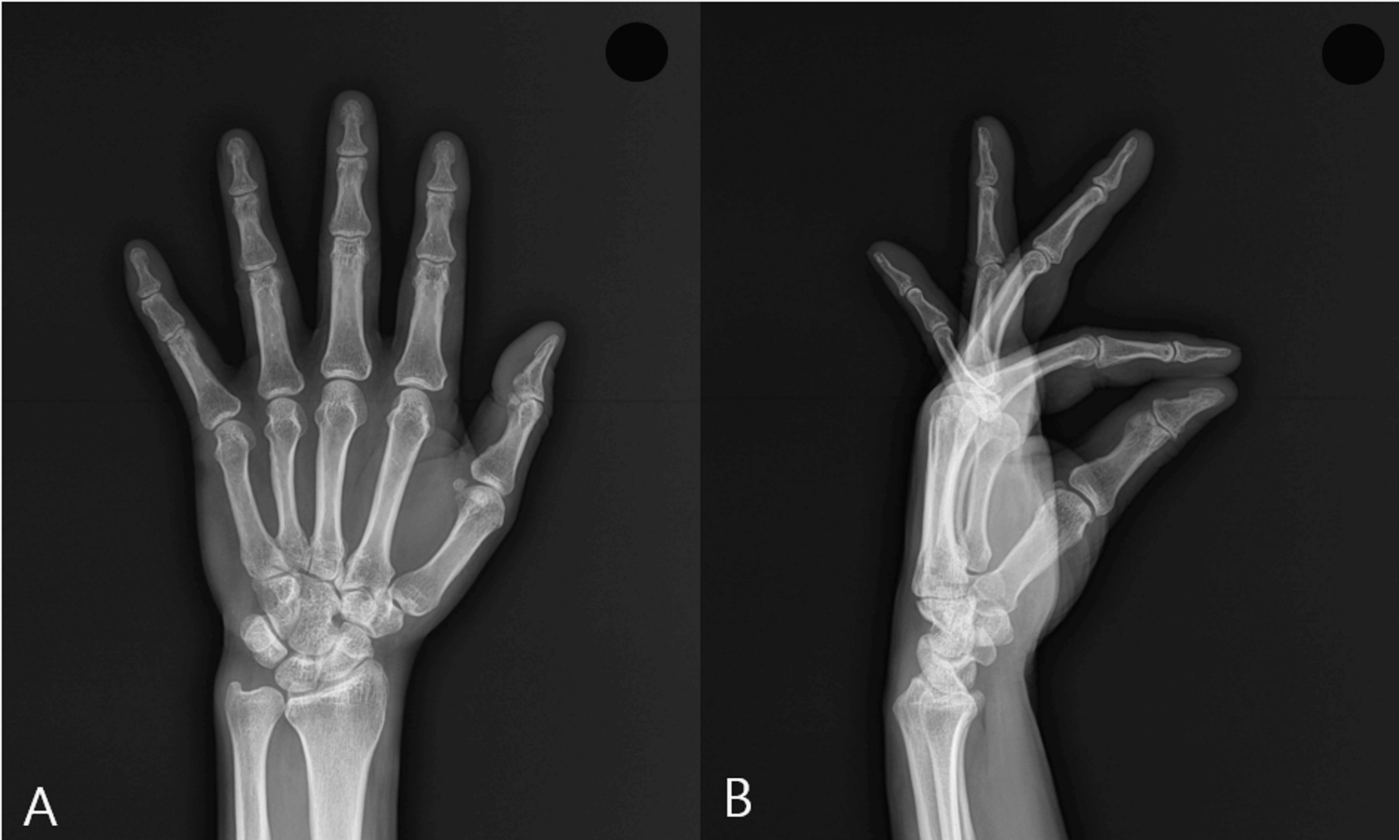
Posteroanterior (PA) (A) and lateral (B) radiographs of the wrist, obtained with the forearm in neutral rotation, demonstrate neutral ulnar variance on the PA view and no evidence of fracture, dislocation, or degenerative change on either view.

Posteroanterior radiography of the wrist in neutral rotation demonstrated neutral ulnar variance without evidence of fracture, dislocation, or degenerative change (Figure 1).

**Figure 2:**
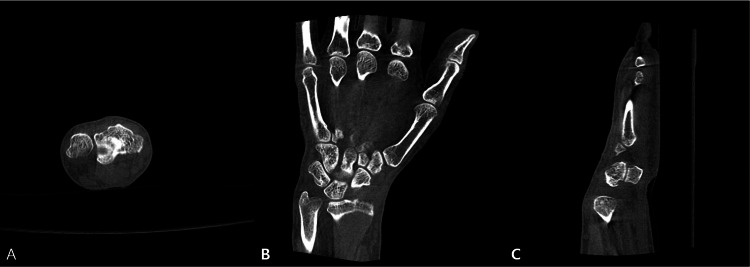
Computed tomography (CT) images of the wrist in the axial (A), coronal (B), and sagittal (C) planes demonstrate no acute fracture or other significant osseous abnormality.

Computed tomography (CT) likewise revealed no acute fracture or other significant osseous abnormality (Figure 2).

**Figure 3:**
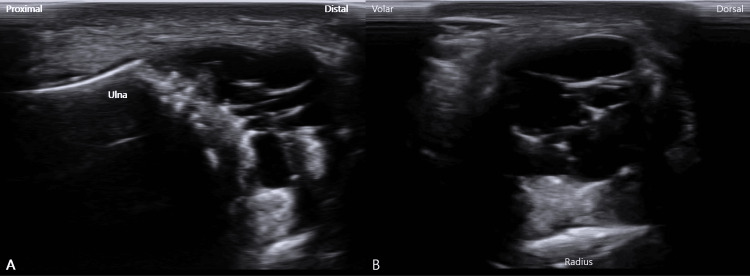
Ultrasound images of the TFCC ganglion cyst. (A) Sagittal ultrasound image demonstrating a well-defined, multiseptated, anechoic ganglion cyst adjacent to the TFCC on the ulnar side of the wrist. (B) Axial ultrasound image showing the multiseptated ganglion cyst in close proximity to the TFCC and distal radioulnar joint. TFCC, triangular fibrocartilage complex

High-resolution ultrasonography clearly delineated the volar, dorsal, and ulnar components of the TFCC and revealed a well-defined, multiseptated, anechoic cystic structure adjacent to the TFCC, consistent with a ganglion cyst (Figure 3).

Intervention

**Figure 4:**
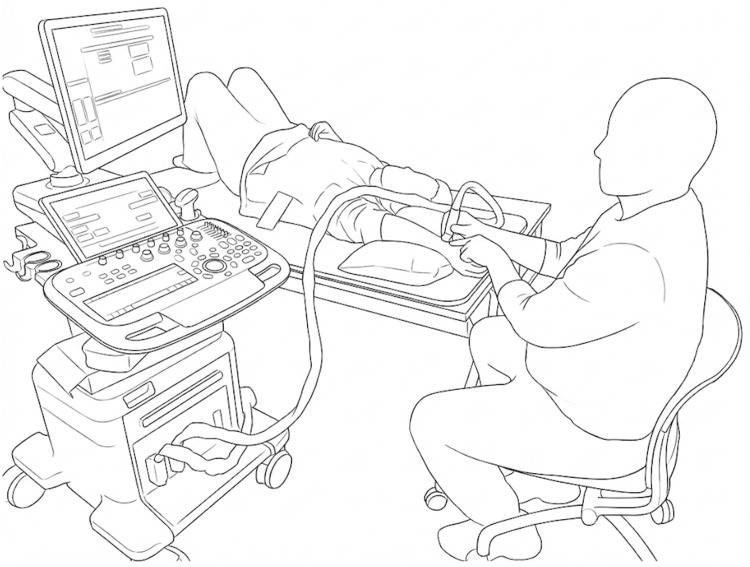
Patient and ultrasound machine positioning for the procedure. The patient is positioned supine with the shoulder abducted and externally rotated in a “statue of liberty” position. The high-frequency linear transducer is placed over the ulnar side of the wrist to visualize the TFCC and ganglion cyst in the short-axis view. Image Credit: Yonghyun Yoon

Under strict aseptic conditions, the patient was positioned supine with the arm abducted and externally rotated in a “statue of liberty” position (Figure 4). Using a high-frequency linear transducer in the short-axis (SAX) view, the ganglion cyst and TFCC enthesis were identified, and the prolotherapy injection was performed using an out-of-plane approach under real-time ultrasound guidance. After local infiltration of 1 mL of 2% lidocaine with a 30-gauge needle, an 18-gauge needle was advanced under real-time ultrasound guidance into the cyst for aspiration.

**Figure 5:**
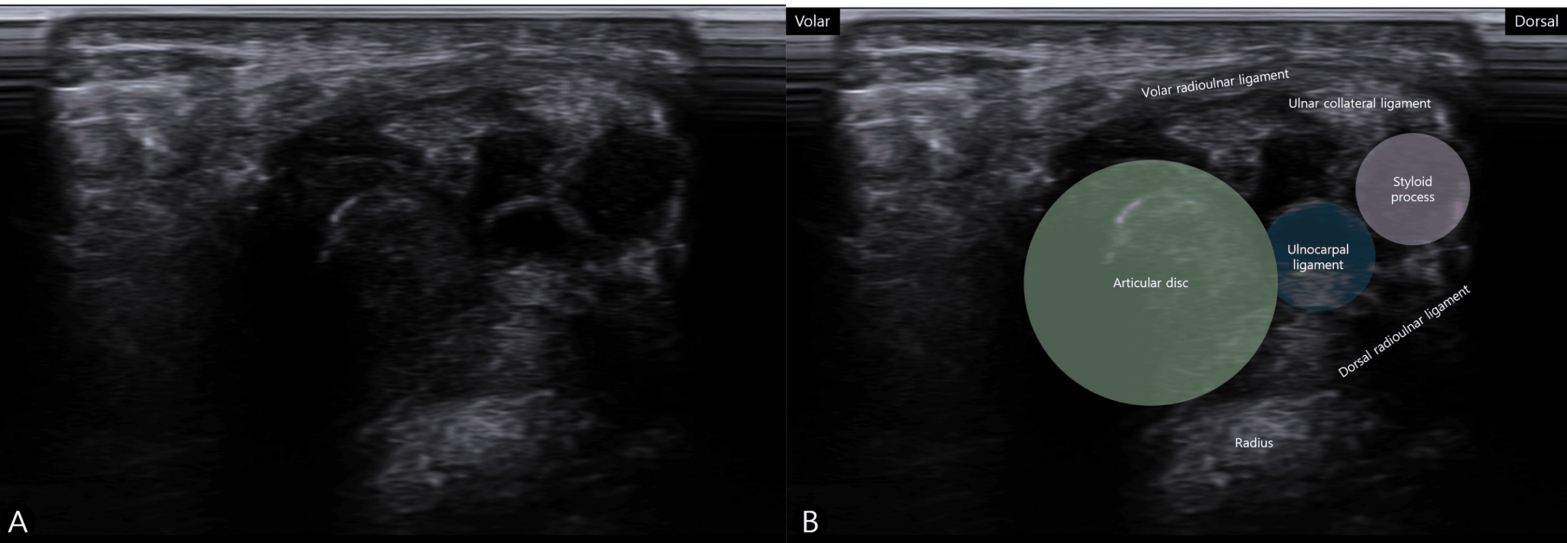
Pre-procedural short-axis ultrasound views used to identify the TFCC enthesis and procedural target prior to ultrasound-guided aspiration and prolotherapy. (A) Reference short-axis ultrasound image obtained before the intervention; no residual ganglion cyst is visible at this stage. (B) The same image as (A) with key anatomical structures labeled and shaded for clarity. TFCC, triangular fibrocartilage complex

Approximately 3 mL of clear, gelatinous fluid was aspirated from the ganglion cyst, with immediate sonographic confirmation of complete cyst collapse. Subsequently, prolotherapy solution (10% dextrose with 0.2% lidocaine) was injected into the TFCC enthesis using an ultrasound-guided out-of-plane approach to stimulate ligamentous healing (Figure 5 and Video 1). Prolotherapy was performed by injecting 0.2 mL of solution per point into multiple points along the TFCC enthesis under real-time ultrasound guidance, with a total injected volume of 5 mL per session. The injections were repeated at two-week intervals for a total of five sessions.

**Video 1:** Real-time short-axis ultrasound demonstrating ultrasound-guided aspiration followed by an out-of-plane prolotherapy injection into the TFCC enthesis (0.2 mL per point; total 5 mL per session). TFCC, triangular fibrocartilage complex

After each procedure, TFCC-stabilizing taping and a ulnar gutter short-arm splint were applied. The patient also participated in a conservative rehabilitation program consisting of weekly physical therapy sessions focused on wrist stabilization and daily home-based isometric exercise training.

Outcome

After the first session, the patient reported a marked reduction in ulnar-sided wrist pain. By the completion of the fifth session, his pain had decreased from a pre-treatment NRS score of 7/10 to 1/10, with resolution of pain during activities of daily living and golf. He was able to resume his usual schedule of business-related golf rounds while wearing supportive taping, without residual pain or functional limitations. At the three-month follow-up, he reported complete pain relief (NRS 0/10).

**Figure 6:**
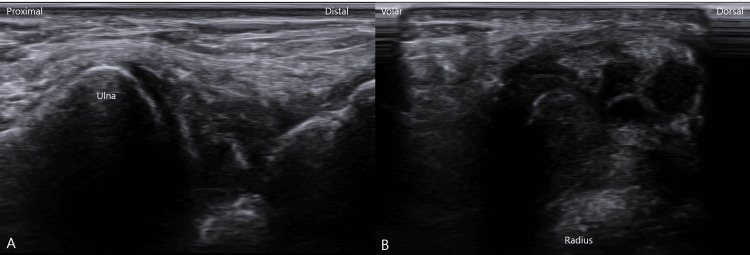
Follow-up ultrasound at three months after treatment. (A) Sagittal ultrasound image demonstrating complete resolution of the previously noted multiseptated ganglion cyst adjacent to the TFCC. (B) Axial ultrasound image confirming absence of the ganglion cyst and preservation of TFCC morphology. TFCC, triangular fibrocartilage complex

At the three-month follow-up visit, physical examination revealed no ulnar fovea tenderness and full, pain-free range of motion. Follow-up ultrasonography demonstrated complete resolution of the ganglion cyst with preservation of TFCC morphology (Figure 6).

## Discussion

This case illustrates two key clinical points. First, it demonstrates that ganglion cysts can arise in association with TFCC pathology, likely as a consequence of chronic, repetitive microtrauma from golf-related wrist loading in a patient whose occupational and social obligations required frequent play. Such repetitive stress may predispose to mucoid degeneration and capsular outpouching, resulting in cyst formation [3].

Second, this case highlights the therapeutic potential of ultrasound-guided, minimally invasive intervention for TFCC-associated ganglion cysts in patients for whom minimizing downtime is particularly important. Ultrasonography was instrumental both for diagnosis and for guiding treatment. Real-time imaging enabled precise needle placement for complete cyst aspiration and accurate delivery of prolotherapy into the TFCC enthesis, thereby maximizing therapeutic effect while minimizing iatrogenic injury [8,9].

Wrist ganglions are commonly treated with aspiration or surgical excision, but recurrence rates remain substantial for both strategies [10]. Ultrasound-guided aspiration has been reported as a safe and potentially effective alternative, particularly for volar lesions close to neurovascular structures. Dextrose prolotherapy is believed to act by inducing a localized inflammatory response that promotes new collagen deposition and strengthens ligamentous structures. Randomized and observational studies suggest that prolotherapy can improve pain and function in chronic wrist and other upper-extremity conditions [6,11-13].

In the present case, the combination of ultrasound-guided aspiration and dextrose prolotherapy provided durable symptom relief without the need for surgery and allowed the patient to maintain an occupationally important level of sports participation. For patients with ulnar-sided wrist pain due to TFCC-associated ganglion cysts, this combined approach may serve as an intermediate option between conservative care alone and operative management.

This report is limited by its single-case design and the relatively short follow-up period of three months. In addition, neither magnetic resonance imaging (MRI) arthrography nor diagnostic wrist arthroscopy was performed, which precludes definitive structural confirmation of TFCC pathology. These investigations were deferred because the patient preferred a minimally invasive approach and demonstrated clear clinical and sonographic improvement following treatment. Larger controlled studies with longer follow-up are warranted to determine generalizability, refine the optimal dosing regimen, and clarify long-term recurrence rates associated with this strategy.

## Conclusions

This case report describes the successful management of a TFCC-associated ganglion cyst using ultrasound-guided aspiration and dextrose prolotherapy in an avid golfer whose occupation required continued participation in the sport. The case underscores the value of ultrasonography as a dynamic diagnostic and interventional tool for ulnar-sided wrist pain and proposes dextrose prolotherapy as a promising adjunctive modality for TFCC pathology. In appropriately selected patients, this minimally invasive technique should be considered as a treatment option before resorting to more invasive surgical procedures.
